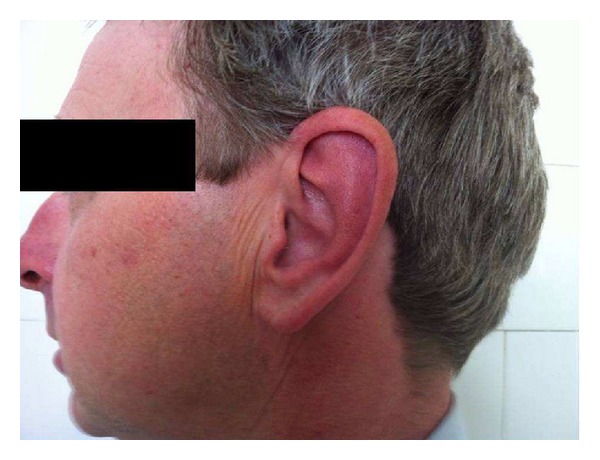# Relapsing Polychondritis in a Patient with Ankylosing Spondylitis Using Etanercept

**DOI:** 10.1155/2014/353782

**Published:** 2014-09-09

**Authors:** Valderilio Feijó Azevedo, Natalia Bassalobre Galli, Alais Daiane Fadini Kleinfelder, Julia Farabolini D'Ippolito, Andressa Gulin Tolentino, Eduardo Paiva

**Affiliations:** ^1^Division of Rheumatology, Department of Clinical Medicine, Federal University of Paraná, 80060-900 Curitiba, PR, Brazil; ^2^Federal University of Paraná, 80060-900 Curitiba, PR, Brazil

## Abstract

Relapsing polychondritis (RP) is an autoimmune disease characterized by recurrent episodes of inflammation and progressive destruction of cartilaginous tissues, especially of the ears, nose, joints, and tracheobronchial tree. Its etiology is not well understood, but some studies have linked its pathophysiology with autoimmune disease and autoantibody production. We described a case of a 46-year-old male patient with ankylosing spondylitis who developed RP after the use of etanercept. Few similar cases have been described in the literature. However, they show a possible association between the use of biological inhibitors of tumor necrosis factor (anti-TNF*α*), which potentially produces autoantibodies, and the development of RP. The treatment was based on data in the literature and included the cessation of biological therapy and the addition of corticosteroids with substantial improvement.

## 1. Introduction

Relapsing polychondritis (RP) is a rare disease characterized by recurring inflammation and destruction of cartilaginous tissues such as the ears, nose, and larynx [1]. Few cases of RP have appeared in the literature, and its pathophysiology is not completely explained, but it is believed that it is an autoimmune event [2]. It is known that around 30% of patients with RP have other concomitant autoimmune diseases, such as systemic lupus erythematosus or Sjögren's syndrome [3]. However, the association with ankylosing spondylitis (AS) is uncommon [4, 5]. Currently, the therapy with antitumor necrosis factor alpha (anti-TNF*α*) is the best alternative to the use of nonsteroidal anti-inflammatories (NSAIDs) for the treatment of AS with predominantly axial symptoms [6, 7]. Although rare, the formation of antibodies and autoantibodies and the development of immune diseases are associated with the use of anti-TNF*α* agents. There is evidence that a small percentage of patients using anti-TNF*α* therapy will develop autoimmune diseases including vasculitis, lupus-like syndrome, and cutaneous psoriatic lesions [8]. Case studies have also revealed a connection between the use of anti-TNF*α* and the development of RP [9].

## 2. Case Report

J. D. is a male, 46 years of age, with clinical presentation of inflammatory low back pain for 10 years, along with stiffness of the lumbar spine in the morning. He began treatment in our ambulatory spondyloarthritis clinic around 1 year earlier, when the definitive diagnosis of AS was made according to the modified New York criteria [10]. The patient was HLA B27-positive and presented with bilateral sacroiliitis in stage III confirmed by conventional radiography.

Despite the continuous use of Ibuprofen 60 mg 8/8 h, sulfasalazine 1 g 12/12 h, prednisone 5 mg/day, and codeine 30 mg 8/8 h for 6 months, the patient's condition worsened, with the inflammatory lower back pain intensifying, synovitis in the right ankle, and unilateral calcaneal enthesitis. At that time, the patient presented a BASDAI score of 6.7 and a CRP of 6.7 mg/dL. According to recommendations from the ASAS group and the consensus of the Brazilian Society of Rheumatology [11, 12], anti-TNF*α* therapy was selected for treatment. Consequently, there was a slow withdrawal of prednisone and an onset of therapy with etanercept at the dose of 50 mg subcutaneous once a week. NSAIDs and sulfasalazine were continued.

Two months after starting etanercept, the patient developed erythema and nasal pain, accompanied by swelling of the left and right ears which did not affect the earlobes (Figure 1). The CRP had fallen to 3.2 mg/dL. ANA and cryoglobulin tests were negative. Other causes of chondritis, such as trauma and infection, were discarded because of the absence of suggestive history. Besides, infectious chondritis usually involves also the earlobe. The presumptive clinical diagnosis of RP was established. The use of etanercept was temporarily suspended and therapy with prednisone 10 mg/day was introduced.

Only three months after the use of anti-TNF*α* was suspended did the patient report improvement in the pain, nasal erythema, and auricular swelling. However, there was a significant worsening of the lower back inflammation and the calcaneal enthesitis. The patient's BASDAI score rose to 7.6, with no significant increase in the RP.

Upon physical examination, there were no nasal alterations. However, mild hyperemia was present in the ears, which was not very painful. We decided to continue corticosteroids therapy and reintroduce anti-TNF*α* therapy with etanercept due to the worsening of the axial symptoms and enthesitis.

After five months of treatment, the patient showed complete improvement of the inflammatory lower back pain, of the arthritis in the ankles, and of the calcaneal enthesitis. The ear and nose symptoms had disappeared. The patient continued to take prednisone 10 mg/day, nimesulide 100 mg 12/12 h, and etanercept 50 mg SC once a week.

## 3. Discussion

The use of anti-TNF*α* drugs has been one of the best alternatives for the treatment of rheumatic diseases which resist treatment with nonsteroidal anti-inflammatories [8]. Etanercept has demonstrated great efficacy in treating the axial symptoms of spondyloarthritis as well as enthesitis and synovitis [7]. Although it is generally well tolerated, studies of etanercept have shown significant adverse effects such as headaches, diarrhea, airway infections, reactivation of latent infections, and, in some cases, the induction of psoriasis and uveitis [7, 9]. Furthermore, patients using anti-TNF*α* may develop autoantibodies such as antinuclear antibodies (ANAs) and anti-double stranded DNA antibodies (anti-DNAds) [8].

Although the etiology of RP is not yet completely understood, it is presumed that it has an autoimmune origin due to its frequent association with autoimmune diseases and with the presence of the human leukocyte antigen (HLA) DR4 [13]. It is also known that anti-collagen antibodies, mainly Type II, can be seen during an acute RP episode; these antibodies are probably the result of the liberation of inflammatory cytokines such as TNF*α*, IL1, and IL6 [14, 15]. The main clinical manifestation of RP is uni- or bilateral auricular chondritis, as seen in our patient. The ear frequently becomes swollen, erythematic, and painful, but the earlobe is not affected [16]. Other important symptoms that may occur in RP are polyarthritis; chondritis of the nasal cartilage, the larynx, and the tracheal cartilage; inflammation of the ocular structures; and involvement of the vestibulocochlear system [2, 3]. However, these symptoms were not reported in this case.

Currently, the use of biological medications has been discussed as part of the treatment for RP, especially in cases when treatment with corticosteroids fails [2, 17, 18], and the use of these molecules has expanded into the treatment of other autoimmune comorbidities [19]. However, two cases of RP during TNF-blocker therapy were reported in Spain in 2011 [20]. Both patients were men with AS, HLAB27 positive, with 45 and 49 years of age, respectively. The first case was characterized by complaining of bilateral pain, erythema, and swelling of the auricle, ocular symptoms, cough, and chest pain after two years of biological therapy with etanercept 25 mg twice weekly. RP was diagnosed based on clinical manifestations; etanercept was stopped and treatment with prednisone 5 mg/day was started. After three months, the treatment with etanercept was reintroduced with no recurrence of RP. The second patient developed bilateral auricular erythema with ocular hyperemia and tearing in both eyes after four years of using infliximab at a dose of 5 mg/kg every 8 weeks. After the diagnosis of RP, anti-TNF treatment was stopped and prednisone 1 mg/kg was started. The patient condition resolved slowly but favorably. The patient remained asymptomatic, but the switch to adalimumab 40 mg every two weeks was necessary because of the recurrence of the axial symptoms.

In the present case report, the hypothesis is that RP was a paradoxical event resulting from the use of anti-TNF*α*, possibly related to the development of autoantibodies which triggered this autoimmune condition. Similar to our patient, none of the patients described in the literature had presented symptoms of RP before the institution of therapy with biologicals. Although 30% of patients with RP have some associated diseases, including vasculitis, diseases of the conjunctive tissues, or autoimmune diseases [21], association with AS is rare [20]. Only three cases associating the two comorbidities without the concomitant use of biologicals have been reported. Pazirandeh et al. described two cases of AS in patients with RP [4], and Bahiri et al. reported a case in a 28-year-old patient who was diagnosed with AS after 5 years of RP [5]. Consequently, the initial suspicion was that RP resulted from the use of etanercept and not a clinical condition associated with AS. According to the few pieces of data in the literature, the response is to interrupt treatment with the biologicals and to begin corticosteroids therapy [22]. With this clinical response, substantial improvement in the diagnosis of chondritis was achieved. Up to the time of this writing, the patient did not develop symptoms compatible with RP, even after reintroducing etanercept therapy. Despite maintaining low-dose corticosteroids therapy, the development of RP and its relation to inhibiting TNF*α* are not completely understood.

## 4. Conclusion

The introduction of anti-TNF*α* drugs into the treatment of RP, ankylosing spondylitis, and other autoimmune diseases has revolutionized the management of patients with active diseases that resist conventional therapy. Nevertheless, the use of these drugs has been associated with the development of immunogenicity and also autoimmunity. The development of autoimmune diseases during treatment with biological drugs is rare but must be recognized promptly to allow appropriate treatment. In the case reported here, the RP diagnosis was eminently clinical and permitted quick and appropriate management of the patient. Although the patient did not develop recurring episodes of polychondritis, other causes of chondritis were discarded. Furthermore, the fact that the auricular symptoms went into remission after the suspension of anti-TNF*α* and the introduction of corticosteroids therapy supports the diagnosis of RP induced by the use of etanercept.

## Figures and Tables

**Figure 1:**